# Potentially inappropriate prescribing and falls-risk increasing drugs in people who have experienced a fall: a systematic review and meta-analysis

**DOI:** 10.1093/ageing/afaf300

**Published:** 2025-10-21

**Authors:** Tim O’Reilly, Jessica Gómez Lemus, Laura Booth, Barbara Clyne, Caroline McCarthy, Kinda Ibrahim, Wade Thompson, Christine McAuliffe, Frank Moriarty

**Affiliations:** School of Pharmacy and Biomolecular Sciences, RCSI University of Medicine and Health Sciences, Dublin, Ireland; School of Pharmacy and Biomolecular Sciences, RCSI University of Medicine and Health Sciences, Dublin, Ireland; School of Pharmacy and Biomolecular Sciences, RCSI University of Medicine and Health Sciences, Dublin, Ireland; Department of Public Health and Epidemiology, School of Population Health, RCSI University of Medicine and Health Sciences, Dublin, Ireland; Department of General Practice, RCSI University of Medicine and Health Sciences, Dublin, Ireland; School of Primary Care, Population Sciences and Medical Education, Faculty of Medicine, University of Southampton, Southampton, UK; National Institute of Health and Care Research (NIHR) Applied Research Collaboration (ARC) Wessex, University of Southampton, Southampton, UK; Department of Anaesthesiology, Pharmacology and Therapeutics, Faculty of Medicine, The University of British Columbia Faculty of Medicine, Vancouver, Canada; School of Pharmacy and Biomolecular Sciences, RCSI University of Medicine and Health Sciences, Dublin, Ireland; School of Pharmacy and Biomolecular Sciences, RCSI University of Medicine and Health Sciences, Dublin, Ireland

**Keywords:** inappropriate, prescribing, prevalence, potentially inappropriate prescribing, falls-risk increasing drug, older people

## Abstract

**Background:**

As certain medications increase risk of falls, it is important to review and optimise prescribing in those who have fallen to reduce risk of recurrent falls.

**Objectives:**

To systematically review evidence on the prevalence and types of potentially inappropriate prescribing (PIP), including falls-risk increasing drug (FRID) use, in fallers.

**Methods:**

A systematic search was conducted in July 2024 in MEDLINE, EMBASE, CINAHL and Google Scholar using keywords for fall events, inappropriate prescribing and FRIDs. Observational studies (cohort, case-control, cross-sectional, before–after) and randomised trials were included. Studies were eligible where participants had experienced a fall and PIP (including FRID use) was reported. Random-effects meta-analyses were conducted to pool prevalence of inappropriate prescribing and mean number of inappropriate prescriptions across studies, with stratified analysis to assess heterogeneity.

**Results:**

Fifty papers reporting 46 studies met the inclusion criteria. All studies assessed FRIDs, and 29 assessed other PIP. The prevalence of PIP at the time of the fall was reported in 43 studies, and the pooled estimate was 68.6% (95% CI 66.1%–71.2%). Amongst 23 studies reporting it, the mean number of inappropriate prescriptions per participant was 2.21 (95%CI 1.98–2.45). The most common FRIDs prescribed were sedatives/hypnotics, opioids, diuretics and antidepressants. Twenty-one studies assessed changes in PIP prevalence post-fall; nine reported decreasing prevalence, with others noting increases/no change/mixed results.

**Conclusion:**

Inappropriate prescribing is highly prevalent amongst fallers, with cardiovascular and psychotropic drugs being the most common. This suggests significant scope to optimise medicines use in these patients to potentially reduce falls risk and improve outcomes.

## Key Points

- The occurrence of a fall can present an opportunity to optimise medications to improve outcomes for a patient.
- Continued use of fall risk increasing drugs is one area of potentially inappropriate prescribing to consider amongst fallers.
- This systematic review identified 46 studies that report prevalence of potentially inappropriate prescribing in fallers.
- In meta-analysis, about two thirds of fallers had potentially inappropriate prescribing.
- There is significant scope for optimising medications amongst fallers, both FRIDs and other potentially inappropriate prescribing.

## Introduction

Medication-related harm is a growing concern and international priority in improving patient safety [1]. Falls are a common adverse outcome, which may have medication-related contributors, [2–4] and are often recurrent. Falls can have a significant and often profound impact on people who experience them, such as reduced mobility or independence, premature admission into long-term care and negative impacts on mental health [5–7]. A fall may present an important opportunity to optimise medication, address potentially inappropriate prescribing or PIP (i.e. prescribing where risks may outweigh benefits for a patient), and reduce the risk of future falls and fractures, or other medication-related adverse outcomes [8, 9]. Amongst fallers, continued use of falls-risk increasing drugs (FRIDs) may be considered potentially inappropriate. As falls are multifactorial, current guidelines recommend that medicines review forms part of multifaceted interventions to reduce falls risk [10]. Adverse events such as falls can prompt reactive medicines review and deprescribing, which occur less frequently in the absence of such triggering events [11].

A previous systematic review quantified FRID use amongst older adults with a fall-related injury and identified 14 studies where prevalence exceeded 65% and no reduction in prevalence was seen after a healthcare encounter [12]. However, this review did not consider other non-injurious falls, nor examine other aspects of PIP, not relevant to falls, where medicines optimisation to address risks other than recurrent falls could be targeted amongst fallers to improve patient outcomes. Understanding the full scope for medicines optimisation amongst all fallers is important to inform targeting of interventions, including which types of drugs are most often implicated in PIP for these individuals.

Therefore, the overall aim of this systematic review is to investigate the prevalence of PIP (including the use of FRIDs) in people with a fall or fall-related injury/event. A secondary aim is to determine the types of drugs most often implicated, and whether the prevalence of PIP changes after a fall.

## Methods

This systematic review was preregistered on PROSPERO (CRD42023417534), conducted in line with JBI guidance, [13] and is reported according to the Preferred Reporting Items for Systematic reviews and Meta-Analyses statement [14].

### Eligibility criteria

The following eligibility criteria were applied:

#### Study type

We included observational studies (cohort, before–after, case-control, cross sectional), and randomised trials where the population studied were people who had fallen. Systematic reviews were excluded; however, any relevant reviews were examined for potentially eligible studies. Other publication types (e.g. conference abstracts, study protocols, commentaries, case series) were excluded.

#### Population

We included studies focusing on adults of any age who experienced a fall (based on any definition). Fall-related events such as fracture and syncope were also included. Studies in which the sample population was people attending a falls clinic or similar were included where 70% or above of study participants had a fall or a fracture or where <70% of participants had a fall or fracture but characteristics of fallers such as age, sex and prevalence of PIP were reported. For case-control studies, only those studies with falls as an outcome that assessed medication exposure within 90 days prior to the fall (indicating likely medication use at the time of fall) were included. Studies were excluded where people who had fallen were an incidental subgroup of the main study population, and not part of the study inclusion criteria.

#### Outcome

We included studies reporting prevalence of PIP, defined using any approach (e.g. validated tools, lists of medication, specific criteria or indicators, local definitions or where medication use was implicitly judged to be inappropriate). This included any FRID use (as continuing FRIDs in fallers was considered potentially inappropriate), other inappropriate prescribing that is deemed to be relevant to fallers (e.g. anticoagulant use due to increased likelihood of bleeding with recurrent falls, omission of bone protection treatment) and any inappropriate prescribing unrelated to falls (including inappropriate omissions). Studies that assessed inappropriate prescribing of only single drugs/drug classes were excluded.

### Information sources and search strategy

MEDLINE (ovid), EMBASE, CINAHL and Google scholar (via Harzing’s Publish or Perish) [15] were searched from inception up to the search date of 5th July 2024. The search strategy (included in Appendices S1–S4 of the Supplementary Data) was developed using a combination of subject headings, keywords and synonyms relating to PIP or FRIDs and falls or fractures. No language or other restrictions were used. Any results which were in a different language were translated using online translation software Deepl (www.deepl.com). Grey literature sources were not searched, as given the subject it was anticipated that such sources would be unlikely to contribute significantly.

### Selection process

Results from each database were combined, and one reviewer deduplicated using Endnote. Remaining results were uploaded to Rayyan, and its deduplication function was used. Pilot title and abstract screening was conducted on 50 records in Rayyan to ensure the eligibility criteria were applied consistently by all reviewers. Following this, each title and abstract was screened independently by two reviewers (of T.O'R., J.G.L., L.B., F.M.). Any disagreements were discussed to reach consensus, or failing this, a third independent reviewer (of T.O'R., C.McA., F.M.) assessed the study. A similar process was followed for full-texts, with a pilot on three studies followed by independent review in duplicate, with disagreements resolved as before.

### Data extraction and data items

A Microsoft Excel sheet was developed to extract data and piloted using three studies. Data were extracted by two independent reviewers (of T.O'R., J.G.L., L.B.). Once complete, a third reviewer (F.M.) checked data for consistency and accuracy across reviewers and studies. Data was extracted on:

- **Study information:** Design, sample size, time frame, geographical location, setting, duration of follow up.
- **Participant information:** Demographics, definition of falls used, proportion with fall/fracture or where reported, proportion of distinct types of falls/fractures.
- **Outcome information**: Definitions of PIP (including FRID use categorised as psychotropic, cardiovascular and other classes), [2–4] time frame prevalence was measured over, prevalence of PIP, prevalence of specific drug classes (involved in PIP/FRID use up to the top 5), and any change in prevalence post-fall.

### Outcomes

The primary outcome measure was prevalence or mean number of PIP amongst fallers. For studies reporting prevalence at multiple time points, the time point closest to the fall was recorded, likely reflecting the medications being taken at the time of the fall. For before-and-after studies and randomised trials, if the prevalence of PIP was reported after a fall, unless explicitly stated that no medication changes had occurred, the prevalence at the latest pre-fall time point was recorded. We extracted the overall percentage prevalence and/or number of individuals affected by PIP, and/or the mean number of PIPs per person and standard deviation. Where an overall prevalence of PIP was not reported, but for example, prevalence based on different tools such as Beers, STOPPFall was reported, the highest prevalence was extracted (being most reflective of the scope for medicines optimisation). The prevalence of PIP at later time points, where reported, was extracted as a secondary outcome to examine change in prevalence post-fall.

### Quality assessment

Study quality was evaluated using the JBI Prevalence Critical Appraisal tool [16]. This assessment was conducted independently by two reviewers (of T.O'R., J.G.L., L.B.) during the data extraction process.

### Data analysis

Characteristics of included studies were summarised descriptively. Meta-analysis of prevalence estimates, and mean number of PIP was performed using the *metan* package in Stata [17]. Heterogeneity can be a concern in meta-analysis of prevalence; the I^2^ statistic and Cochran’s Q test are reported despite limitations, [18] and this was supplemented by stratifying analyses by study characteristics (i.e. setting, inclusion of fractures, time frame for prevalence measurement, PIP definition, and as a *post hoc* analysis, categories of mean/median age). A meta-regression was conducted for PIP prevalence including these characteristics, publication year, and mean/median age.

## Results

**Figure 1:**
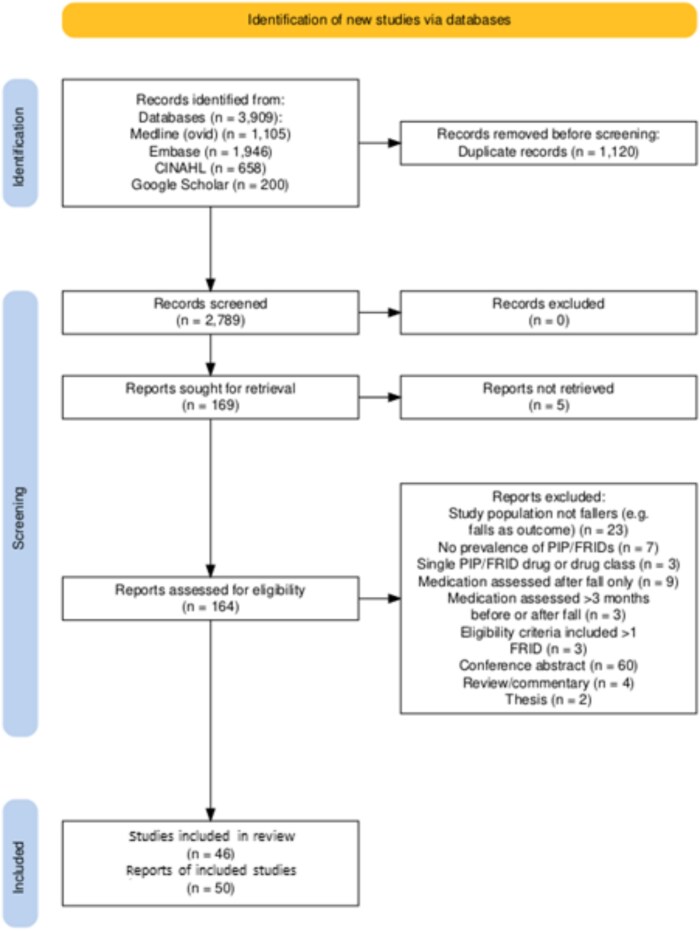
PROSPERO flow diagram of included/excluded publications.

From 3909 records identified, after deduplication 2789 titles/abstracts were screened, and 164 publications underwent full-text review (see Figure 1) [19]. Overall, 50 publications, relating to 46 studies, met the eligibility criteria. Two studies reported relevant results in two publications each, [20–23] while another study had its results published across three different publications [24–26].

### Quality assessment

Included studies were mostly of high quality (Appendix S5 of the Supplementary Data), however 20 studies had small sample sizes (*n* < 200), while nine studies did not describe the study setting in detail.

### Study characteristics

Of the 46 studies published between 2007 and 2024 (Table 1), 19 were cohort studies, 11 were cross-sectional studies, 10 were case-control studies, 4 were before-and-after studies, 1 was a quasi-experimental study, and one was a randomised controlled trial. Studies were performed in the USA (*n* = 9, one conducted in the USA/Mexico border region), Japan (*n* = 5), Sweden (*n* = 4), France (*n* = 3), United Kingdom (*n* = 3), Australia (*n* = 2), Czech Republic (*n* = 2), Ireland (*n* = 2), Netherlands (*n* = 2), Taiwan (*n* = 2), with one study each from Austria, Belgium, Brazil, Canada, Colombia, Denmark, Finland, Germany, Malaysia, New Zealand, Norway and Spain.

**Table 1:** Descriptive characteristics of included studies, listed by year of publication.

Study, year, location	Study design, setting, time frame	Fall-related inclusion (% with event), sample size	Source of PIP definition (specific tool used)	PIP definition categories					Time frame of PIP prevalence measurement
				FRID CVD	FRID Psy	FRID Oth	PIP Fall	PIP Oth	
van der Velde *et al.* 2007 [42] Rotterdam, Netherlands	Prospective cohort study, Geriatric Outpatient Clinic, ≥65 years 19 months, Apr 2003–Nov 2004	Fall (100%) *n* = 139	Literature source(s)	Yes	Yes	Yes	No	No	Time point, admission to Geriatric Outpatient Department
Sjöberg *et al.* 2010 [43] Gotenburg, Sweden	Prospective cohort study, Hospital, ≥65 years 8 months, Mar–Oct 2008	Hip fracture (100%) *n* = 100	Literature source(s)	Yes	Yes	Yes	Yes	No	Time point, admission
Kragh *et al.* 2011 [44] Sweden	Retrospective cohort study, Hospital ≥60 years 12 months, 2006	Hip fracture (100%) *n* = 2043	Literature source(s)	Yes	Yes	Yes	Yes	No	Period, 6 months pre-hip fracture
Freeland *et al.* 2012 [45] South Carolina, USA	Retrospective cross-sectional study, Outpatient internal medicine clinic, ≥65 years 13 months	Fall (100%) *n* = 116	Literature source(s) (Beers 2003)	Yes	Yes	Yes	No	No	Time point, at time of the fall
Sjöberg Lönnbro Belfrage studies 2013 [24–26] Gotenburg, Sweden	Randomised controlled trial, Department of Geriatrics (Hospital), aged ≥65 years 6 months, Apr–Sep 2009	Hip fracture (100%) *n* = 132	Literature source(s) (STOPP/START v1 and FRID list from Swedish National Board of Health and Welfare)	Yes	Yes	Yes	Yes	Yes	Time point, admission
Bennett *et al.* 2014 [46] Sydney, Australia	Prospective cohort study, Tertiary Hospital (aged care, orthopaedic, general medicine department), aged ≥60 years 10 months, Jun 2012–Mar 2013	Fall (100%) *n* = 204	Literature source(s)	Yes	Yes	Yes	No	No	Time point, admission
Hohmann *et al.* 2014 [47] California, USA	Prospective cohort study, Trauma Centre (level 2), aged ≥65 years 15 months, Aug 2009–Oct 2010	Fall (100%) *n* = 112	Literature source(s) (Beers 2003 and 2012, STOPP/START v1)	Yes	Yes	Yes	Yes	No	Time point, admission
McMahon *et al.* 2014 [48] Ireland	Before and after study, Hospital Emergency Department, aged ≥70 years 4 years, 2007–2010	Fall (100%) *n* = 1016	Literature source(s) (STOPP v1 and Beers 2012)	Yes	Yes	Yes	Yes	Yes	Period, 12 months pre-fall
Rojas-Fernandez *et al.* 2015 [39] Ontario, Canada	Retrospective cross-sectional study, Retirement Facility, aged ≥65 years 12 months, Jul 2009–Jul 2010	Fall (100%) *n* = 105	Literature source(s)	Yes	Yes	Yes	Yes	No	Time point, List of current medications at time of fall
Munson *et al.* 2016 [27] USA	Retrospective cohort study, Community dwelling individuals, no age restriction 5 years, 2007–2011	Fracture (100%) *n* = 168 133	Literature source(s)	Yes	Yes	Yes	Yes	No	Period, 120 days pre-fall
De Winter *et al.* 2016 [49] Flanders, Belgium	Case-control study, Traumatology ward (Hospital), aged ≥75 years 7 months, Nov 2012–Jun 2013	Fall-related fracture (33.5%) *n* = 182	Literature source and expert opinion	Yes	Yes	Yes	Yes	No	Time point, admission medicines reconciliation
Bambina *et al.* 2017 [50] France	Prospective cross-sectional study, Hospital (orthopaedic and trauma department), aged ≥65 years 6 months, Nov 2015–Apr 2016	Fall-related fracture (100%) *n* = 89	Not stated	Yes	Yes	Yes	No	No	Time point, admission
Blachman *et al.* 2017 [51] New York, USA	Retrospective cross-sectional study, Hospital, aged ≥65 years 1 year, 2012	Fall (100%) *n* = 328	Literature source(s)	No	Yes	Yes	No	No	Time point, 24 hours pre-fall
Komagamine *et al.* 2017 [52] Japan	Retrospective cohort study, Hospital, aged ≥65 years 2 years, Jan 2015–Dec 2016	Hip fracture (100%) *n* = 164	Literature source and expert opinion (Beers 2015)	Yes	Yes	Yes	Yes	No	Time point, admission
Marvin *et al.* 2017 [53] London, England	Retrospective cohort study, Hospital Emergency Department, aged ≥70 years 2 months, Feb–Mar 2015	Fall (100%) *n* = 100	Literature source and expert opinion (STOPP v2)	Yes	Yes	Yes	No	No	Time point, admission
Ryan-Atwood *et al.* 2017 [54] Victoria, Australia	Case-control study, Specialist Trauma Centre, aged ≥65 years 2 years, Jul 2013–Jun 2015	Fall (73.8%) *n* = 646	Literature source(s)	Yes	Yes	Yes	No	No	Time point, admission medicines reconciliation
Beunza-Sola *et al.* 2018 [55] Navaree, Spain	Prospective cohort study, Tertiary level hospital, aged ≥65 years 4 months, Mar–Jun 2016	Fall-related fracture (100%) *n* = 252	Literature source(s) (STOPP v1)	Yes	Yes	Yes	No	No	Time point, admission
Lawson *et al.* 2018 [56] Texas/Mexico Border Community	Retrospective cross-sectional study, Community dwelling older adults, aged ≥55 years Not reported	Fall (100%) *n* = 99	Literature source(s) (Beers 2012)	Yes	Yes	Yes	Yes	Yes	Time point, baseline study data collection, within 1 month of fall
Machado-Duque studies 2018 [20, 21] Colombia	Case-control study, Community Pharmacy dispensing records of those with a hip fracture recorded in the General System of Social Security in Health of Colombia, aged ≥65 years 1 year, Jan–Dec 2015	Fracture (33.3%) *n* = 900	Literature source and expert opinion (Salahudeen extended Anticholinergic Risk Scale)	Yes	Yes	Yes	Yes	No	Period, 30 days prior to index date
Early *et al.* 2019 [57] USA	Case-control study, Inpatient, outpatient, and ambulatory care (Medicare database), aged ≥65 years 3 years, Jan 2013–Sep 2015	Fall (6.6%) *n* = 1 678 037	Literature source(s)	Yes	Yes	Yes	No	No	Period, 90 days pre-fall
Gleich *et al.* 2019 [58] Germany	Retrospective cohort study, Trauma centre (level 1), aged ≥70 years 2 months, Jul 2016–Sep 2016	Hip or humerus fracture (100%) *n* = 95	Literature source(s) (STOPP/START v2 and PRISCUS list)	Yes	Yes	Yes	Yes	Yes	Time point, admission
Maly *et al.* 2019 [29] South Bohemia, Czech Republic	Prospective cross-sectional study, Hospital wards of four hospitals (Internal, Nursing, surgical, Psychiatry, Pneumology, Rehab), aged ≥18 years 12 months, Jan–Dec 2017	Fall (100%) *n* = 288	Expert opinion/review	Yes	Yes	Yes	No	Yes	Time point, 24 hours pre-fall
Morin *et al.* 2019 [59] Sweden	Case-control study, Non-elective Hospital or Emergency Department admission, aged ≥70 years 12 months, Jan–Dec 2013	Fall (50%) *n* = 49 609	Literature source(s)	Yes	Yes	Yes	No	No	Period, 7 days pre-fall
Walsh *et al.* 2019 [34] Ireland	Before and after study, GP practice records of patients hospitalised with fall, aged ≥65 years 5 years, Jan 2011–Jan 2016	Fall, fracture, or syncope (100%) *n* = 927	Literature source(s) (STOPP/START v2)	Yes	Yes	No	Yes	No	Period, 12 months prior to the index hospitalisation
Andersen *et al.* 2020 [41] Denmark	Retrospective cross-sectional study, Hospital, aged ≥65 years 24 weeks (2017)	Hip fracture (98.5%) *n* = 200	Literature source(s) (STOPP v2)	Yes	Yes	Yes	Yes	Yes	Time point, admission
de Ruiter *et al.* 2020 [60] Alkmaar, Netherlands	Retrospective cohort study, Falls and Syncope Clinic, aged ≥65 years 5.5 years, Nov 2011–Jun 2016	Fall or syncope (100%) *n* = 374	Literature source and expert opinion (Dutch revised version of STOPP/START)	Yes	Yes	Yes	Yes	No	Time point, pre-review at Falls and Syncope Clinic
Michalcova *et al.* 2020 [40] Brno, Czech Republic	Retrospective cross-sectional study, Nursing home, Geriatric care unit in hospital, aged ≥60 years 2 years, Jan 2016–Dec 2017	Fall (100%) *n* = 188	Expert opinion/review	Yes	Yes	Yes	No	No	Period, during the in-patient stay
Nagai *et al.* 2020 [36] Japan	Retrospective cohort study, Hospital, aged ≥65 years 4 years, Oct 2014–Dec 2018	Fracture (100%) *n* = 253	Literature source(s) (STOPP-J)	Yes	Yes	Yes	Yes	Yes	Time point, admission
Tiihonen *et al.* 2020 [61] Lahti, Finland. Kouvola Finland	Prospective cohort study, Hospital, aged ≥50 years 12 months, Oct 2015–Oct 2016	Fracture (100%) *n* = 245	Literature source(s)	No	Yes	No	No	No	Time point, admission
Weeda *et al.* 2020 [62] USA	Retrospective cohort study, Emergency Department and Hospital, aged ≥65 years 3 months, Jan–Mar 2019	Fall (100%) *n* = 292	Literature source(s) (Beers 2019)	No	Yes	Yes	No	No	Time point, admission
Nagai *et al.* 2021 [37] Japan	Retrospective cohort study, Single hospital, aged ≥65 years 5 years, Oct 2014–Aug 2019	Fracture (100%) *n* = 170	Literature source(s) (STOPP-J)	Yes	Yes	Yes	Yes	Yes	Time point, admission
Shibasaki *et al.* 2021 [63] Matsudo, Japan	Retrospective cohort study, Rehabilitation Unit (Hospital), aged ≥65 years 4 years, Jan 2015–Dec 2018	Fracture (100%) *n* = 217	Literature source(s) (Beers 2015 and START v2)	Yes	Yes	Yes	Yes	Yes	Time point, admission
Escórcio Brito Rêgo *et al.* 2021 [64] Maranhão, Brazil	Retrospective cross-sectional study, Hospital Municipal de Imperatriz (HMI), aged ≥60 years 12 months, Jan 2019–Jan of 2020	Hip fracture (100%) *n* = 29	Literature source(s) (Beers 2019 and Lexi-Interact® database used for potential drug–drug interactions.)	Yes	Yes	Yes	Yes	Yes	Period, during the hospitalisation period of the patients (average 10.5 days)
Chiam *et al.* 2022 [33] Kuala Lumpur	Retrospective cross-sectional study, Falls and Syncope Clinic, aged ≥65 years 5 years, Aug 2014–Jun 2019	Fall or syncope (100%) *n* = 421	Literature source and expert opinion (STOPP/START v2)	Yes	Yes	Yes	Yes	Yes	Time point, medication list from first visit to falls clinic
Hart *et al.* 2022 [65] Washington, USA	Prospective cohort study, Outpatient visits, hospitalizations and ED visits for a fall-related injury, aged ≥65 years 10 years, 1994–2014	Fall (100%) *n* = 1516	Literature source(s)	No	Yes	Yes	No	No	Time point, admission
Li *et al.* 2022 [66] Taipei City, Taiwan	Case-control study, Various hospital wards, aged ≥50 years 18 months, Apr 2018–Sep 2019	Fall (16.6%) *n* = 786	Literature source(s) (Beers 2019)	Yes	Yes	Yes	Yes	Yes	Time point, medications prescribed on the day of the fall
Morishita *et al.* 2022 [32] Tokyo, Japan	Case-control study, Medical University Hospital, aged ≥18 years 12 months, Jan–Dec 2016	In-hospital falls (50%) *n* = 894	Literature source(s)	No	Yes	No	No	No	Time point, day before the fall
Cox *et al.* 2023 [67] England	Prospective cohort study, Fracture Clinic, aged ≥65 years 12 months, Mar 2019–Mar 2020	Fracture (100%) *n* = 100	Literature source(s) (STOPPFall)	Yes	Yes	Yes	No	No	Time point, medicines at baseline recruitment from fracture clinic
Léguillon *et al.* 2023 [68] France	Before–after-control-impact (BACI) study, Geriatric perioperative care units, aged ≥75 years 18 months, Control Feb–Sep 2019 and Intervention Feb–Sep 2020	Hip fracture (100%) *n* = 209	Literature source(s) (STOPP/START v2 and Medication Appropriateness Index (used to assess both PIMs and PPOs))	Yes	Yes	Yes	Yes	Yes	Time point, admission
Fluck *et al.* 2023 [69] South-West Surrey, England	Retrospective cross-sectional study, NHS hospital, aged ≥60 years 10 years, Apr 2009–Jun 2019	Hip fracture (100%) *n* = 1105	Literature source(s) (Ageing Brain Program’s Anticholinergic Cognitive Burden score)	Yes	Yes	Yes	Yes	No	Time point, admission
Podesser *et al.* 2023 [70] Austria	Case-control study, Geronto-Psychiatric Hospital, aged ≥65 years 22 months, Jun 2016–Mar 2018	Fall (50%) *n* = 74	Literature source(s) (Anticholinergic Drug Scale)	No	Yes	No	Yes	No	Time point, 24 hours pre-fall
Yang *et al.* 2023 [31] Taiwan	Case-control study, Changhua Christian Hospital, aged ≥20 years 5 years, Jan 2017–Dec 2021	Fall (20%) *n* = 4260	Literature source(s) (Swedish National Board of Health and Welfare)	Yes	Yes	Yes	No	No	Period, 3 days pre-fall
Henriksen *et al.* 2023 [30] Tønsberg, Norway	Before and after study, Regional hospital, aged ≥18 years 10 months, Control Jun–Aug 2018 and Intervention Sep 2018–Apr 2019	Hip fracture (100%) *n* = 108	Literature source(s) (Norwegian translation of STOPP criteria v2)	Yes	Yes	Yes	Yes	Yes	Time point, admission
Selman Casey studies 2023 [22, 23] North Carolina, USA	Prospective cohort study, Hospital Emergency Department, aged ≥65 years 16 months, Aug 2020–Dec 2021	Fall (100%) *n* = 572	Literature source(s) (STEADI-Rx list and the 2019 AGS Beers criteria.)	Yes	Yes	Yes	Yes	No	Time point, admission medicines reconciliation
Corvaisier *et al.* 2024 [28] Angers, France	Quasi-experimental study, Geriatric day hospital dedicated to older patients with a recent history of falls, no age restriction 2 years, Jan 2020–Dec 2021	Fall (82%) *n* = 233	Literature source and expert opinion	Yes	Yes	Yes	Yes	No	Time point, admission
O’Leary *et al.* 2024 [71] Christchurch, New Zealand	Case-control study, Metropolitan subacute hospital, aged ≥65 years 9 months, Jul 2019–Feb 2020	Fall (50%) *n* = 400	Literature source(s) (Drug burden index (DBI))	Yes	Yes	Yes	Yes	No	Time point, admission

The most common age restriction was ≥65 years (*n* = 26); all but six studies required participants to be ≥50 years. Two studies had no age restriction, [27, 28] although one of these included geriatric day hospital patients, and four required patients to be adults (aged ≥18/≥20 years) [29–32]. Mean or median age of participants was ≥65 years in all studies, and was ≥80 years in 33 studies, while females accounted for most fallers in all studies except five (where between 53.9% and 62.1% of fallers were male).

Twenty-three studies included participants with falls or fall events, 20 studies focused on fractures, while two studies focused on falls and syncope, [30, 33] and one on falls, fractures and syncope [34]. Falls and fractures were defined in different ways across the studies. Twenty-two studies described fall events using different free-text definitions, which were developed with reference to literature. Twelve studies defined falls/fractures using International Classification of Diseases codes [35]. Nine studies did not report how falls/fractures were defined. One study used the AO/OTA fracture and dislocation classification compendium to define a fracture [36]. One study used the visual SQ method to define vertebral fractures as proposed by Genant *et al.* [37, 38] One study used the French Society of Geriatrics and Gerontology criteria for serious falls [28].

Thirty-three studies included inpatients and/or attendees at emergency departments, six studies included attendees of an outpatient clinic, five studies included community-dwelling individuals with a record of a fall across inpatient and outpatient/ambulatory care settings (or unspecified settings), and one study each included individuals from a retirement facility, [39] and from both a nursing home and hospital geriatric care unit [40].

Numbers of participants ranged from 29 to 1 678 037. In 34 studies, 100% of their participants had a fall, one study of 200 consecutive hip fracture patients identified that 98.5% of participants had a fall [41], while another study in a geriatric day hospital included 82% of participants with a recent fall history [28]. In the 10 case-control studies, the percentage of fallers included in the study ranged from 6.6% to 73.8%.

### Definitions of potentially inappropriate prescribing

In assessing PIP amongst fallers, all studies considered FRIDs (in some cases as part of a validated tool for PIP). Three studies assessed only a single category of FRIDs (psychotropics), four studies assessed two categories of FRIDs (three studies assessed psychotropic and other categories, and one study assessed psychotropic and cardiovascular categories), while the remaining 39 studies assessed all three categories. Overall, 46, 42 and 40 studies assessed psychotropic, other and cardiovascular FRIDs, respectively. Twenty-nine studies also assessed other forms of PIP, of which 28 assessed PIP relevant to falls, and fourteen studies assessed non-fall relevant PIP.

Thirty-six studies cited literature source(s) for their PIP definition (including FRIDs), seven cited literature source(s) and expert opinion, two studies referred to expert opinion/review alone, and one did not state the source. Twenty-six studies reported the use of a validated tool for defining PIP. Seventeen studies used a single tool, most often the Beers criteria (five studies) or the STOPP criteria (six studies), and one study each used the STOPPFall criteria, Drug Burden Index, Anticholinergic Cognitive Burden score, Anticholinergic Drug Scale, the Salahudeen extended Anticholinergic Rating Scale, and the Swedish National Board of Health and Welfare indicators. Eleven studies used combinations of tools, most often STOPP/START (*n* = 3), with one study each using other combinations of tools (STOPP/START and Beers criteria, STOPP/START and the PRISCUS list, STOPP/START and Sweden’s FRID list, STOPP/START and Medication Appropriateness Index, STOPP and Beers, START and Beers, Beers and Lexi-Interact®, and Beers and the STEADI-Rx list).

Thirty-six studies assessed prevalence of PIP at a time point, most often reported as at admission (25 studies) followed by at the time of or on the day of the fall (seven studies), while 10 studies assessed PIP prevalence over a time period, ranging from 3 days to 12 months pre-fall.

### Overall prevalence of potentially inappropriate prescribing

A measure of the prevalence of PIP was reported by all included studies, with the percentage of participants with PIP reported in 43 studies, and the mean number of PIP occurrences per participant was reported or calculable in 23 studies (Table 1). Twenty studies reported both measures.

**Figure 2:**
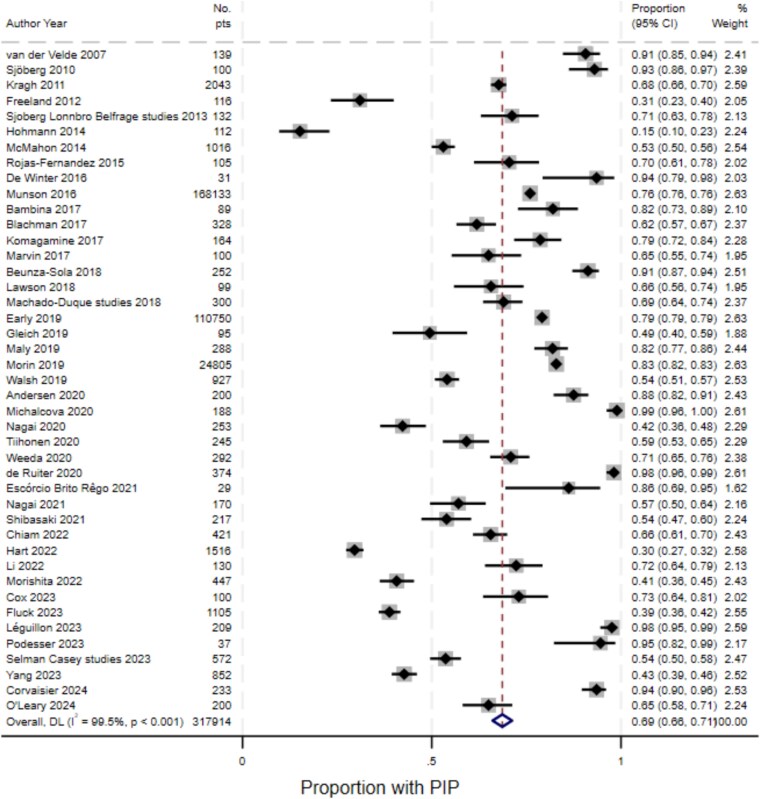
Forest plot for random-effects meta-analysis of studies reporting prevalence of potentially inappropriate prescribing.

The prevalence ranged from 15% to 99%. Across 317 914 participants in included studies, the pooled prevalence (Figure 2) was estimated at 68.6% (95%CI 66.1%, 71.2%), however, there was substantial between-study heterogeneity (I^2^ 99.5%, Cochran’s Q *P* < .001). Heterogeneity was examined in sub-group analysis, and this was not explained by any aspect of study design or included participant characteristics, except for mean/median age of participants, where studies with participants aged ≥85 years on average having a prevalence of 83.5% (95%CI 76%–91%) (see Appendix S6 of the Supplementary Data). In meta-regression, none of the included study or participant characteristics were significantly associated with prevalence (see Appendix S7 of the Supplementary Data).

**Figure 3:**
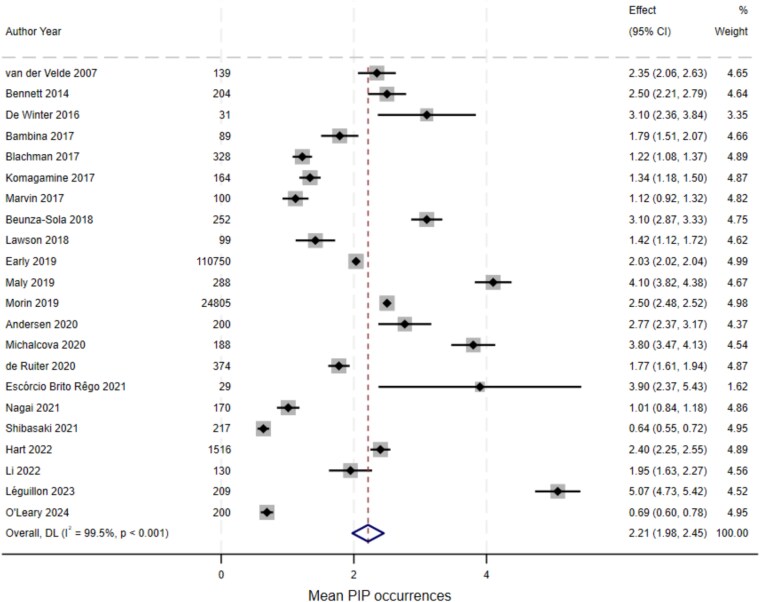
Forest plot for random-effects meta-analysis of studies reporting mean number of potentially inappropriate prescribing occurrences per participant.

In the 23 studies reporting the mean number of PIP occurrences per participant, this ranged from 0.6 to 5.1. Overall two studies reported means less than 1, nine between 1 and 2, five between 2 and 3, and seven between 3 and 4. The pooled mean (Figure 3) was estimated at 2.21 (95%CI 1.98, 2.45) PIP occurrences per participant, however there was substantial between-study heterogeneity (I^2^ 99.5%, Cochran’s Q *P* < .001). Overall prevalence was not explained by study design or included participant characteristics, with the exception of whether PIP was assessed at a time point (mean 2.12, 95%CI 1.65, 2.68) or over a period (mean 2.77, 95%CI 2.38, 3.16), *P* = .034 for Cochran’s Q statistic for between-group heterogeneity (see Appendix S8 of the Supplementary Data).

### Prevalence of individual potentially inappropriate prescribing drugs

**Figure 4:**
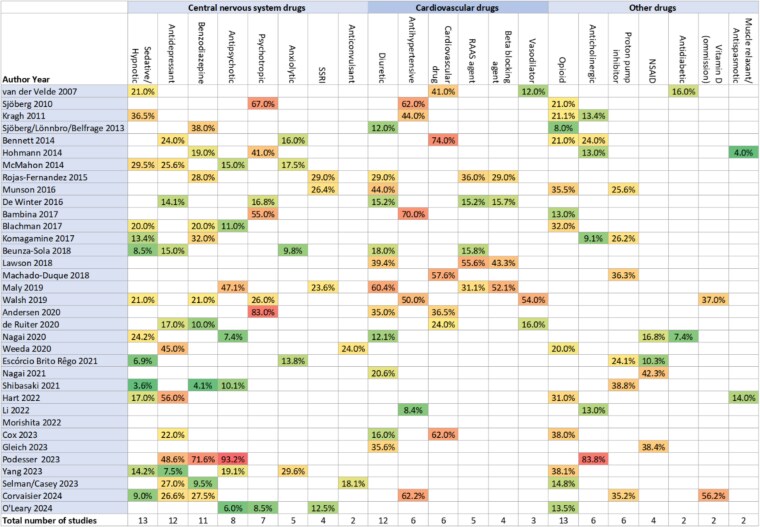
Heat plot of the prevalence of the top 5 drug classes reported as involved in potentially inappropriate prescribing (including FRIDs). ‘Note: Drug classes only reported amongst the top 5 classes in fewer than two studies were omitted.’

Thirty-five studies reported on the prevalence of different drug classes implicated in PIP, and up to the top five most prevalent are reported in Figure 4. Sedative/hypnotic drugs and opioids were reported in 13 studies each, with the percentage of participants prescribed them in the ranges of 3.6%–36.5% and 8%–38.1%, respectively. The next most frequently reported in 12 studies each were antidepressants (7.5%–56%) and diuretics (12%–60.4%).

### Changes in potentially inappropriate prescribing

Twenty-one studies reported prevalence of PIP after the fall (Appendix S9 of the Supplementary Data), with 11 of these reporting prevalence at discharge (and two of these also followed up at 6 and 12 months). The remaining ten reported prevalence at 1, 3, 4, 6 and/or 12 months post-fall. An increase in PIP post-fall was identified in five studies [24, 34, 44, 55, 62]. Three studies identified no change, while a further five identified mixed results (i.e. increases, decreases, and/or no change across different drug classes or follow-up periods). The remaining nine studies identified reductions in PIP post-fall.

## Discussion

Overall, this systematic review of 46 studies identified that PIP is common amongst fallers internationally. Most studies involved ED/hospitalised patients 65 years and over, all evaluated FRID use while approximately two-thirds also assessed other forms of PIP. The pooled PIP prevalence was 68.6%, with fallers having 2.2 PIP occurrences on average. The most commonly reported drug classes were opioids, sedatives/hypnotics, antidepressants and diuretics. Fewer than half of studies (*n* = 21) evaluated change in PIP over time, and just under half of these found a reduction in PIP post-fall. Few studies examined non-falls-related PIP, which makes it challenging to compare the most prevalent types of PIP amongst fallers with previous research in general populations, and suggests a need for further research.

Opioids, sedatives/hypnotics, antidepressants and diuretics were the medication classes most frequently reported as falls-risk increasing or potentially inappropriate in fallers, corresponding to the three major categories of FRIDs. Diuretics have been shown in the literature to be a leading cause of medication induced orthostatic hypotension and volume depletion in older patients, [72] which may explain their high prevalence amongst fallers. Opioids can cause sedation and cognitive impairment, with pharmacokinetic changes in older adults amplifying these effects and increasing falls risk [73]. Sedative and hypnotic medications such as benzodiazepines are amongst the most prescribed psychotropic medication, and particularly with chronic use leading to dependence and tolerance, they can cause sedation, impaired balance and potentially cognitive impairment, all risk factors for falls [74]. The broad clinical domains of these medications underlines the importance of a holistic assessment of prescribing appropriateness amongst fallers, especially those with multiple chronic conditions.

Deprescribing long-term medications can be difficult and may explain why medications deemed to be potentially inappropriate may be continued. Various deprescribing guidelines are available, including for benzodiazepines and opioids, [75, 76] while a diuretics guideline is in development [77]. These provide evidence based recommendations to support decision-making, covering how to identify when and how to reduce or stop medications which are no longer necessary or where potential risks outweigh benefits. An adverse event such as a fall may provide strong support to consider deprescribing, weighed against potentially beneficial effects of the medication [78]. It may be clinically appropriate to continue some potentially inappropriate prescriptions after a fall where the long-term benefits outweigh the anticipated harms, and so some level of post-fall FRID use may be appropriate. Evidence to date on the effect of deprescribing interventions for falls prevention has been mixed, [79, 80] and further robust evaluations of the impact of such interventions as part of multifactorial strategies amongst patients with falls would be beneficial.

Notably there was substantial between-study heterogeneity both in the proportion of people with falls who had PIP, and mean PIP occurrences. Prevalence was partly explained by age group, with studies with a mean or median age of 85 years over having higher prevalence, which is particularly concerning given this age group likely most at risk of adverse consequences from FRIDs, PIP, as well as falls and fractures. For mean PIP occurrences, this was partly explained by whether PIP was measured at a time period or over a period, indicating a time point may not capture the full complexity of medication use [81]. However, substantial heterogeneity remained amongst studies, and this could not be explained by reported study-level characteristics examined in meta-regression. Studies used a variety of different definitions for PIP and reported these differently (e.g. total prevalence or prevalence per validated tool). Even amongst studies using the same tool, such as STOPP/START or Beers criteria, different versions of these or adaptations to the local context (e.g. due to lacking the data required for application or medications not available in a jurisdiction) may contribute to heterogeneity in prevalence estimates. Future studies should ensure that any such adaptations are clearly reported as some studies did not clearly describe which criteria were applied or omitted. Considering that there are a multitude of factors that contribute to PIP, it is likely that other characteristics not measured or reported at both study- and participant-level, e.g. prevalence of particular conditions and multimorbidity, frailty or number of medicines, may further explain heterogeneity.

### Strengths and limitations

A strength of this review is the inclusive approach for defining falls and related-events, populations and settings, and PIP, yielding a comprehensive synthesis of research in this area, which complements other more focused evidence synthesis such as the review of falls-related injuries, which included only 14 studies, all of which were included in this review [12]. The review protocol was preregistered and followed methodological guidance for systematic reviews of prevalence studies. Limitations include the focus on peer-reviewed literature, given the likely low contribution of grey literature sources to the topic, however potentially relevant research may be omitted. Heterogeneity in prevalence was high, which may reflect differences in study design, populations, drug classes used to define PIP/FRIDs and other aspects not examined in stratified analyses [18]. Within the review timeframe, it was not feasible to contact study authors to obtain information not reported. For example heterogeneity in how drug and drug class prevalence was reported across studies impeded further statistical analysis for specific drugs/classes.

### Implications

Future studies on this topic should adopt a standardised approach to recording and reporting medication use at the individual drug and drug class levels, which would enhance the evidence base for targeted approaches to address FRIDs amongst fallers. Similarly, more comprehensive assessment of not just FRIDs, but also other forms of PIP amongst fallers should be considered in further research, including a full description of the criteria applied. Further research is also needed to determine the extent to which medication review and optimisation occurs after a fall, as fewer than half of included studies reported on this.

Depending on the healthcare context, a falls admission may provide an opportunity to review and optimise medicines use in general, e.g. where a fall-related admission triggers a comprehensive geriatric assessment. The recent World Guidelines for Fall Prevention and Management recommend medication review and appropriate deprescribing of FRIDs as part of multifactorial falls prevention [10]. However a recent systematic review on the effectiveness of medication review and deprescribing interventions as a single intervention in falls prevention identified wide heterogeneity in interventions and did not identify a significant effect (although this was amongst all populations, not specifically people with an existing fall) [80]. Likelihood of benefit may be increased by focusing on individuals taking FRIDs with strong evidence of an impact on falls or other factors which predict falls risk [82, 83].

## Conclusion

The high prevalence amongst fallers of PIP, including FRID use, identified in this review suggests significant scope for medicines optimisation in this group, particularly amongst the oldest old. This could focus on falls risk reduction and improving prescribing appropriateness more generally, however the evidence that this occurs routinely is limited and mixed. Improved targeting of deprescribing interventions to address key FRID classes, as part of multifactorial falls prevention strategies may ultimately reduce future falls and improve patient outcomes.

## Supplementary Material

Supplementary_materials_afaf300
